# Effect of topical isopropyl unoprostone on macular atrophy progression in eyes with exudative age-related macular degeneration

**DOI:** 10.1097/MD.0000000000006422

**Published:** 2017-03-24

**Authors:** Chieko Shiragami, Masahiro Miyake, Atsushi Fujiwara, Yuki Morizane, Akitaka Tsujikawa, Ayana Yamashita, Fumio Shiraga

**Affiliations:** a Department of Ophthalmology, Kagawa University Faculty of Medicine, Kagawa; b Department of Ophthalmology and Visual Sciences, Kyoto University Graduate School of Medicine, Kyoto; c Department of Ophthalmology, Okayama University Graduate School of Medicine, Dentistry and Pharmaceutical Sciences, Okayama, Japan.

**Keywords:** a big potassium channel, age-related macular degeneration, endothelin 1, fundus autofluorescence, isopropyl unoprostone, macular atrophy

## Abstract

**Background:**

To evaluate the efficacy and safety of topical isopropyl unoprostone (IU) in treating macular atrophy in age-related macular degeneration (AMD) patients.

**Methods:**

Fifty-two AMD patients with macular atrophy were included and randomly assigned (1:1) to the treatment (topical 0.15% IU) or placebo group. Subjects used study eye drops 3 times a day for 54 weeks. The macular atrophy was documented on fundus autofluorescence photographs and measured using RegionFinder. The enlargement rate of macular atrophy and the changes in visual acuity were examined statistically between baseline and 54 weeks.

**Results:**

Forty-eight subjects were included in the analyses because 4 subjects withdrew from the study. The differences between the IU and placebo groups in mean and median area of macular atrophy were not statistically significant at baseline. The baseline median lesion size of macular atrophy was 2.33 mm^2^ in the IU group and 1.63 mm^2^ in the placebo group (*P* = 0.51). The intergroup difference in the enlargement ratio of macular atrophy (21 ± 15% in the IU group and 111 ± 96% in the placebo group) was statistically significant (*P* < 0.001). Additionally, visual acuity tended to improve over baseline in the IU group. No serious adverse events were observed.

**Conclusions:**

Topical IU therapy is safe and effective for treating macular atrophy in AMD patients.

## 1 Introduction

Geographic atrophy (GA) is the atrophic late-stage manifestation of dry, age-related macular degeneration (AMD), and represents approximately 20% of all late-stage AMD cases.^[1,2]^ It is characterized by the development of atrophic areas that enlarge steadily over time and are associated with a corresponding absolute scotoma.^[3–5]^ Early symptoms of dry AMD include drusen and pigmentary alteration, and progressive loss of retinal pigment epithelial (RPE) cells followed by photoreceptors; the different clinical forms can be identified with autofluorescence imaging.

Recently, antivascular endothelial growth factor (VEGF) therapy has emerged as a major breakthrough in treating exudative AMD, but a treatment protocol that halts or reverses macular atrophy progression has not yet been developed.^[6]^ However, protein complex formation, RPE hypertrophy, and cell death, the first events in macular atrophy, and also in GA in dry AMD, have been observed after anti-VEGF treatment.^[7,8]^ Intravitreal ranibizumab (IVR; Lucentis, Novartis Pharma AG, Basel, Switzerland; and Genentech, Inc., South San Francisco, CA) is 1 of the most widely used agents for treating exudative AMD. Two previous studies evaluated IVR monotherapy over a 2-year period and reported macular atrophy incidences of 25.8%^[9]^ and 71.4%.^[10]^ Schmitz-Valckenberg et al^[11]^ demonstrated that semiautomated software (RegionFinder, version 1,5,0; Heidelberg Engineering, Heidelberg, Germany) for quantifying geographic macular atrophy associated with dry AMD, which uses confocal scanning laser ophthalmoscopy fundus autofluorescence (FAF) imaging, yielded both accurate and reproducible results. The FAF signal is very low, or even extinguished, in areas of macular atrophy, because healthy and aging/suffering RPE cells contain lipofuscin, the dominant fluorophore in the retina. Therefore, FAF images are useful for macular atrophy detection and sizing.

Isopropyl unoprostone (IU; R-tech Ueno, Tokyo, Japan) is a metabolized form of prostaglandin F2α, a chemically synthesized docosanoid (22-carbon basic skeleton). Topical 0.12% and 0.15% IU ophthalmic solutions were approved for clinical use in Japan in 1994 and in the United States in 2001, respectively. ^[12–14]^ Topical IU decreases intraocular pressure by increasing the uveoscleral and trabecular outflow. The route of penetration of IU into the posterior ocular tissues is by direct diffusion through the eye^[15]^ or through the ocular blood circulation via the nasal and conjunctival vascular systems. Topical IU has been used to treat glaucoma and ocular hypertension for many years, but clinical studies have shown that topical IU use can increase retinal and choroidal blood flow.^[16,17]^ One study demonstrated that human choroidal blood flow improved after ocular instillation of 1 to 4 drops of 0.12% IU, q30 min for a total of four doses, after the blood flow was decreased by an injection of endothelin 1 (ET-1) intravenously.^[17]^

The IU molecule is a big potassium (BK, maxi-K) channel activator, not a prostaglandin F receptor agonist.^[18]^ The BK channel only reaches the activation threshold during cell depolarization and/or when intracellular Ca^2+^ levels are high. ^[18,19]^ It has been demonstrated in vivo that the IU molecule protects retinal ganglion cells against ET-1-induced neuronal injury through extracellular signal-regulated kinase phosphorylation in a dose-dependent manner.^[20]^ In an in vivo model, IU inhibits glutamate stimulation and opens maxi-K channels, which are potassium channels that reach an activation threshold only during depolarization and/or at high intracellular Ca^2+^ concentrations. The resultant large efflux of K^+^ hyperpolarizes the cell, thereby closing voltage-gated Ca^2+^ channels and limiting neuronal damage by decreasing influx of intracellular Ca^2+^. Additionally, an in vitro study demonstrated that IU inhibits apoptosis of rat retinal neuroglial progenitor R28 cells.^[21]^ More recently, IU was shown to protect mouse cone photoreceptors and human RPE cells against oxidative stress and light-induced damage through BK channel activation.^[22]^

Clinical studies of patients with retinitis pigmentosa (RP) showed that topical administration of 0.12%^[23]^ and 0.15%^[24]^ IU may lead to improved retinal sensitivity. Reduced choroidal blood flow was shown to occur in patients with RP and was suggested to play a role in photoreceptor damage.^[25]^ The authors suggested that choroidal circulation improvements might preserve photoreceptors in RP patients. In support of an impaired choroid circulation, an increase in plasma ET-1 levels, reflective of decreased foveal choroidal blood flow, has been reported in some RP patients.^[26]^ Interestingly, multiple instillations of IU were shown to partially block ET-1-induced vasoconstriction of human choroidal vessels.^[17]^ In AMD patients with macular atrophy, the improvement of choroidal circulation might be important for preventing the secondary death of photoreceptors.^[27–29]^ In this study, we examine the safety and efficacy of topical 0.15% IU in preventing macular atrophy enlargement in eyes with exudative AMD.

## 2 Patients and methods

### 2.1 Study design and participants

The Institutional Review Board/Ethics Committee of the Kagawa University, Faculty of Medicine, approved this study, which was registered in the University Hospital Medical Information Network Clinical Trials Registry (ID: UMIN000007881). This prospective, placebo-controlled, pilot study was conducted between May 2, 2012 and November 8, 2013 in the Department of Ophthalmology of Kagawa University Hospital. Written informed consent was obtained from each participant before any study procedures or examinations were performed and study conduct adhered to the tenets of the Declaration of Helsinki.

All participants were diagnosed with exudative AMD and met all of the following criteria: age between 40 and 85 years; a decimal best-corrected visual acuity (BCVA) ≥20/200; detectable macular atrophy on FAF imaging and fundus examination; and a dry macula for at least 6 months after treatment with IVR and/or photodynamic therapy (PDT) for exudative AMD. The exclusion criteria included the following: signs of exudative changes, including intraretinal or subretinal fluid, pigment epithelial detachment, or hemorrhage; presence of any other retinal disorder potentially confounding the clinical assessment (eg, diabetic retinopathy, retinal vein occlusion, or retinal artery occlusion); myopia greater than 6 diopters; any previous treatment with direct laser photocoagulation; presence of significant media opacities (eg, cataract or corneal opacity); and pregnancy. All patients had been treated at least once with a variety of medications before enrollment.

### 2.2 Randomization and masking

Randomization was performed by alternately assigning patients to the IU and placebo groups as they entered the study. The IU group received topical 0.15% IU ophthalmic solution (R-tech Ueno, Tokyo, Japan), and the placebo group received the drug vehicle (R-tech Ueno). Both preparation bottles and caps were identical so that the subjects remained masked to group assignment.

### 2.3 Definition of macular atrophy

Macular atrophy was defined as a hypofluorescent area detected by FAF imaging, which included fibrovascular scarring, drusen, and surrounding RPE atrophy, in this study.

### 2.4 Primary and secondary outcome measures

The primary outcome measure was the percent of macular atrophy enlargement from baseline to 54 weeks. The secondary endpoint was the proportion of patients with preserved BCVA over the 54-week study period. The mean measurement of the enlargement rate of macular atrophy was the most important indicator for confirming the effect of topical IU on macular atrophy in eyes with exudative AMD. The enlargement rate of macular atrophy was defined as the area final/area baseline.

### 2.5 Study procedures and examinations

All subjects were instructed to administer one study drop in the study eye at 5-minute intervals each morning, afternoon, and night. The participants were examined at the clinic at enrollment (baseline) and at 4, 8, 12, 24, 36, and 54 weeks. A review of ocular and systemic symptoms and adverse events was conducted at each visit. All follow-up, examinations and evaluations were performed by a single physician (C.S.).

All patients underwent the following examinations at each study visit: FAF imaging, decimal BCVA, and optical coherence tomography (OCT; Spectralis, Heidelberg Engineering, Germany). The presence of macular atrophy was determined using digital red-free fundus photographs (TRC-50DX; Topcon, Tokyo, Japan) and FAF images (Spectralis HRA + OCT), obtained at each visit.

Retinal FAF images were obtained with an excitation light bandwidth of 488 nm in a 30° × 30° field of view centered on the macula. The images had a resolution of 768 × 768 pixels. The RegionFinder software was used to identify the areas of macular atrophy and define the borders. Detection and measurement of macular atrophy was performed by a single examiner (A.F.) who was masked to treatment assignment. Fundus color photographs and FAF photographs were used to detect and measure the size of macular atrophy.

### 2.6 Statistics

This study was designed to have 90% power for detecting a difference in the enlargement rate of macular atrophy of 15% from baseline to 54 weeks using a 2-sided, unpaired *t* test and a significance level of 0.05. Assuming a 10% dropout rate, the required sample size was calculated to be 26 patients per study group. For this calculation, the standard deviation of enlargement rate of macular atrophy was assumed to be 15 based on the previous reports.^[1,30,31]^

Baseline values and proportions were compared between groups using unpaired *t* tests for continuous variables and chi-square or Fisher exact tests (if any frequency was <5) for categorical or binomial variables, respectively. Because the data for the area of macular atrophy were not normally distributed, but were skewed to the right, we performed a log transformation before performing the *t* test. For the same reason, the median and quantiles are presented in addition to the mean and standard deviation for the area of macular atrophy.

Primary and secondary outcomes were evaluated using unpaired *t* tests. Longitudinal analyses using a generalized estimating equation were also conducted to evaluate the influence of IU on the area of macular atrophy and the logarithm of the minimum angle of resolution (logMAR) visual acuity. Potential confounders, including age, sex, and AMD subtype, were included in modeling. The timing of evaluations (ie, 4, 8, 12, 24, 36, and 54 weeks) was treated as a continuous variable.

All analyses were performed using SAS statistical software (version 9.3, SAS Institute, Cary, NC), except for the generalized estimating equation, which was performed using software R (ver. 3,0,2 R Foundation for Statistical Computing, Vienna, Austria). Statistical significance was defined as *P* < 0.05.

## 3 Results

The 52 enrolled patients were randomized into 2 groups of 26 patients each. One subject in the placebo group chose to withdraw from the study, and 3 subjects (1 placebo, 2 IU) had their study drops discontinued by the investigator because of adverse events (see below). Because the reason for the absence was unrelated to the response, we did not employ the imputation analyses to avoid the bias generated by imputation. Therefore, IU efficacy was determined using data from the 48 subjects (24 placebo, 24 IU) who completed the 54-week trial.

Forty-eight eyes of 48 patients (18 women, 30 men) were included in this 54-week-long prospective study. All participants presented with unilateral macular atrophy and were followed up at 3-month intervals using a standardized protocol. Baseline demographic and ocular data are summarized in Table 1. No statistically significant differences were found between the placebo and IU groups.

**Table 1:**
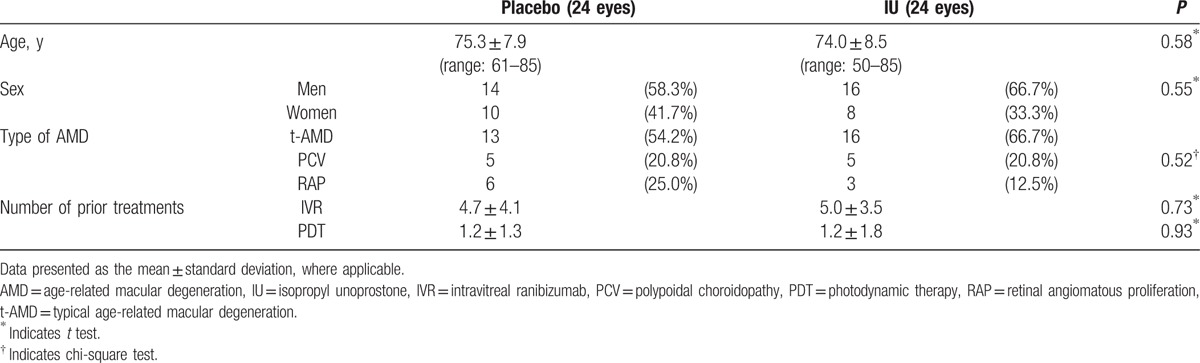
Baseline demographic and ocular data for participants.

### 3.1 Enlargement rate of macular atrophy

**Figure 1:**
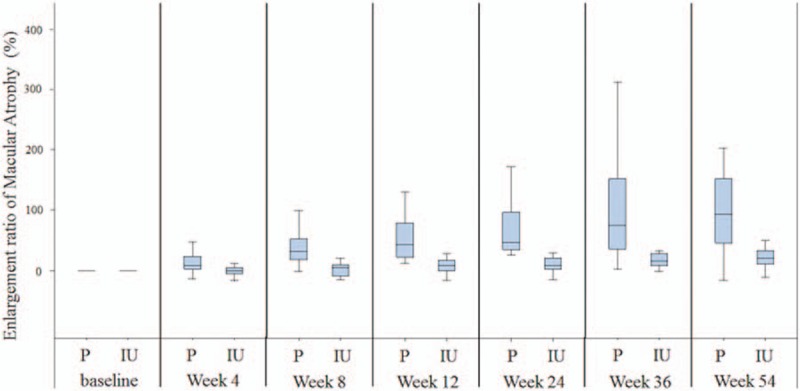
Enlargement ratio of macular atrophy over the 54-week follow-up period. At 54 weeks, the macular atrophy enlargement ratio of the placebo and IU placebo groups were 111 ± 96% and 21 ± 15%, respectively (*P* < 0.001, *t* test). The mean is the horizontal line, the boxes are the first/third quartile, and the bars are the SEM. IU = isopropyl unoprostone, P = placebo, SEM = standard error of mean.

The difference in the enlargement ratio of macular atrophy (21 ± 15% in the IU group, and 111 ± 96% in the placebo group) between the groups was statistically significant (*P* < 0.001, *t* test). This was also true at each interim time point examined (Fig. 1). In the IU group, the size of macular atrophy relative to baseline was −2 ± 12%, 1 ± 10%, 8 ± 16%, 11 ± 14%, and 13% ± 19% at 4, 8, 12, 24, and 36 weeks, respectively. This was significantly lower (all *P* < 0.001, *t* test) than in the placebo group, which had a size of macular atrophy relative to baseline of 13 ± 31%, 40 ± 31%, 56 ± 44%, 70 ± 49%, and 97 ± 73% at 4, 8, 12, 24, and 36 weeks, respectively.

### 3.2 Change in lesion area as measured on fundus autofluorescence images

The differences between the IU and placebo groups in the mean and median area of macular atrophy, as measured on the FAF images, were not statistically significant at baseline. The baseline median size of the macular atrophy lesion was 2.33 mm^2^ (first quartile: third quartile = 1.28:3.73) in the IU group and 1.63 mm^2^ (first quartile: third quartile = 1.02:3.61) in the placebo group (*P* = 0.51). The average and quartile area of the macular atrophy lesion for both the IU and placebo groups are shown in Table 2. The increase in lesion area, as quantified using FAF evaluation, was not significantly different between groups.

**Table 2:**
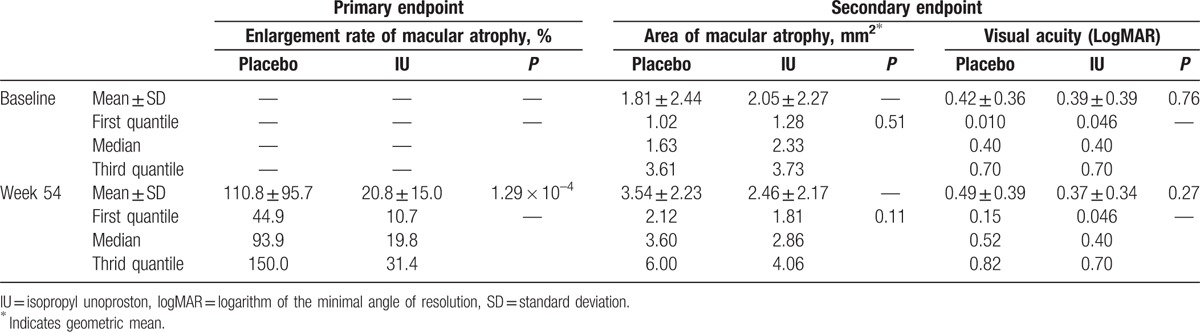
Characteristics of macular atrophy.

**Figure 2:**
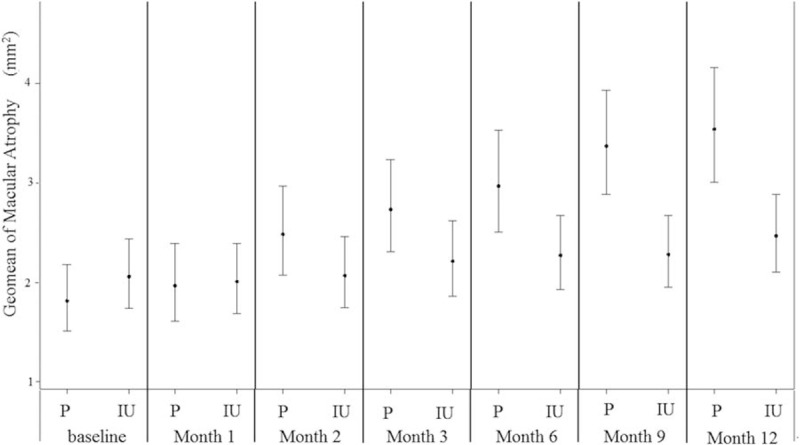
Change in mean area of macular atrophy over the 54-week follow-up period. At baseline, the geometric mean area of macular atrophy (represented by absent autofluorescence) was 1.81 ± 2.44 mm^2^ and 2.05 ± 2.27 mm^2^ in the placebo and IU groups, respectively. At 54 weeks, the median area of macular atrophy was 3.60 and 2.86 mm^2^ in the placebo and IU groups, respectively. The difference between the areas of macular atrophy between groups was not statistically significant (*P* = 0.119). IU = isopropyl unoprostone, P = placebo.

The median area of macular atrophy in the IU group was 2.33, 2.24, 2.42, 3.01, 2.92, 3.27, and 2.86 mm^2^ at baseline and 4, 8, 12, 24, 36, and 54 weeks, respectively. The median area of macular atrophy in the placebo group was 1.63, 1.60, 2.32, 3.73, 2.51, 2.45, and 3.60 mm^2^ at baseline and 4, 8, 12, 24, 36, and 54 weeks, respectively (Fig. 2).

### 3.3 Mean visual acuity

**Figure 3:**
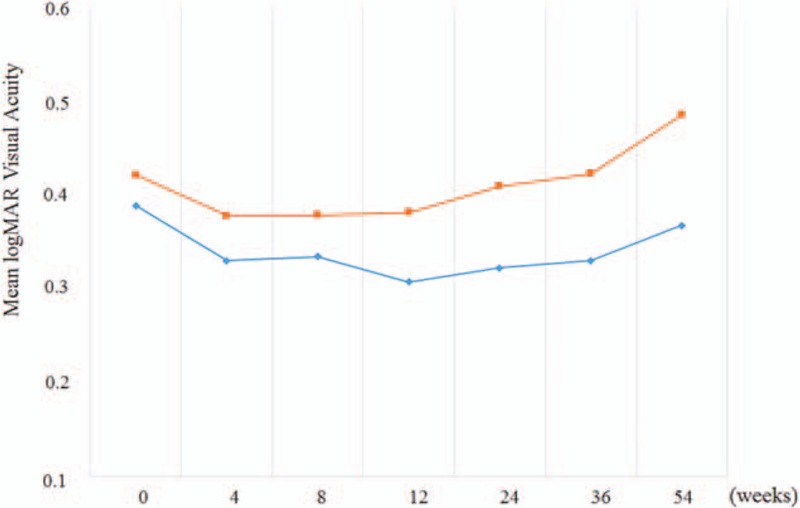
Change in visual acuity over the 54-week follow-up period (red line = placebo group, blue line = isopropyl unoprostone group). At 12 months, the mean logarithm of the minimum angle of resolution (logMAR) best-corrected visual acuity (BCVA) was 0.42 ± 0.36 (Snellen equivalent = 20/40) and 0.39 ± 0.39 (20/35) in the placebo and isopropyl unoprostone group, respectively (*P* = 0.76, *t* test). Though the trend was striking and the difference was clinically significant, the difference in BCVA between the groups at 12 months was not statistically significant (*P* = 0.27, *t* test).

Mean logMAR BCVA in the placebo and IU groups was 0.42 ± 0.36 (Snellen equivalent = 20/40) and 0.39 ± 0.39 (20/35) at baseline, a slight difference that was not statistically significant (*P* = 0.76). The logMAR BCVA progressively declined in the placebo group and was 0.38 ± 0.33 (20/38), 0.41 ± 0.36 (20/39), 0.42 ± 0.33 (20/41), and 0.49 ± 0.39 (20/43) at 3, 6, 9, and 12 months, respectively. In contrast, mean logMAR BCVA remained stable during the study period and was 0.31 ± 0.32 (20/32), 0.32 ± 0.35 (20/33), 0.33 ± 0.33 (20/34), and 0.37 ± 0.34 (20/36) at 3, 6, 9, and 12 months, respectively. Although, the difference between groups in the BCVA was not statistically significant at any time point examined (*P* = 0.43, 0.40, 0.33, and 0.27 at 3, 6, 9, and 12 months, respectively), BCVA tended to improve over baseline in the IU group (Fig. 3).

### 3.4 Factors related to the area of macular atrophy and visual acuity changes

The longitudinal analysis after adjusting for potential confounders is summarized in Table 3. Interaction term of time and IU were significantly associated with the area of macular atrophy (*P* < 0.001), and logMAR BCVA (*P* = 0.018), indicating that IU significantly impacted the time course of the area of macular atrophy and visual acuity changes. However, the other interaction terms were not significantly associated with BCVA. Particularly, the subtype of retinal angiomatous proliferation (*β* = −0.612, *P* = 0.017) was associated with inhibiting the enlargement of area of macular atrophy.

**Table 3:**
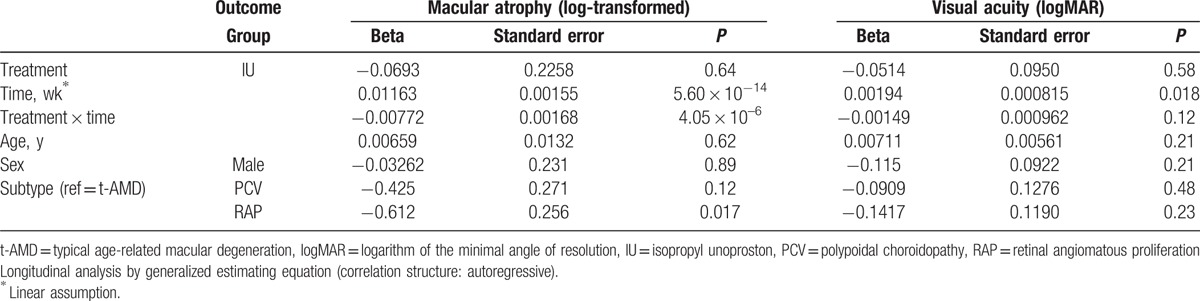
Linear regression for longitudinal data.

### 3.5 Case Reports

#### 3.5.1 Seventy-four-year-old man with typical AMD (IU group)

**Figure 4:**
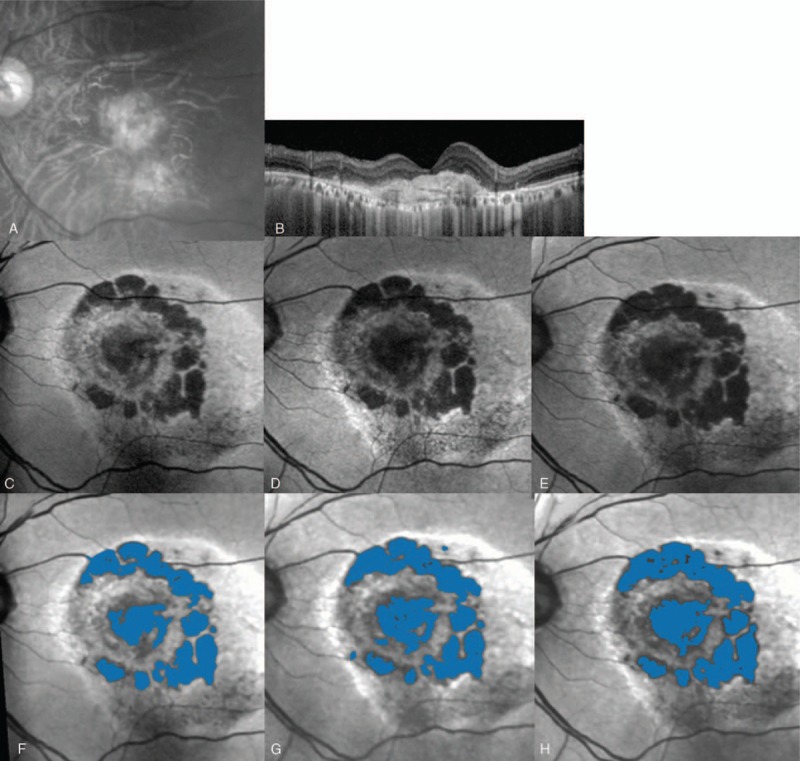
Case 1: A 74-year-old man with typical age-related macular degeneration in the isopropyl unoprostone group. He had received 10 prior intravitreal ranibizumab injections, 3 of which were in conjunction with photodynamic therapy. A gray-scale fundus photograph (A) showing a fibrous scar surrounding an area of retinal pigment epithelial (RPE) atrophy. An optical coherence tomography image (B) showing no exudative lesion. Fundus autofluorescence photographs at baseline (C), 24 weeks (D), and 54 weeks (E) showing the hypofluorescent macular atrophy lesion. The area of macular atrophy (shaded blue) was 2.9 mm^2^ at baseline (F), 5.4 mm^2^ at 24 weeks (G), and 5.8 mm^2^ at 54 weeks (H). The enlargement rate of macular atrophy was 18%.

A 74-year-old man with typical AMD was placed in the IU group (Fig. 4). He had received 3 prior PDT treatments and 10 prior IVR, and had a dry macula at the time of enrollment. At baseline, the gray-scale fundus photograph showed macular atrophy (Fig. 4A), and OCT showed RPE loss (Fig. 4B). To quantify macular atrophy, FAF photographs were taken at baseline (Fig. 4C, F), 24 weeks (Fig. 4D, G), and 54 weeks (Fig. 4E, H). The enlargement rate of macular atrophy was 18% over the 54-week study.

#### 3.5.2 Seventy-nine-year-old woman with typical AMD (placebo group)

**Figure 5:**
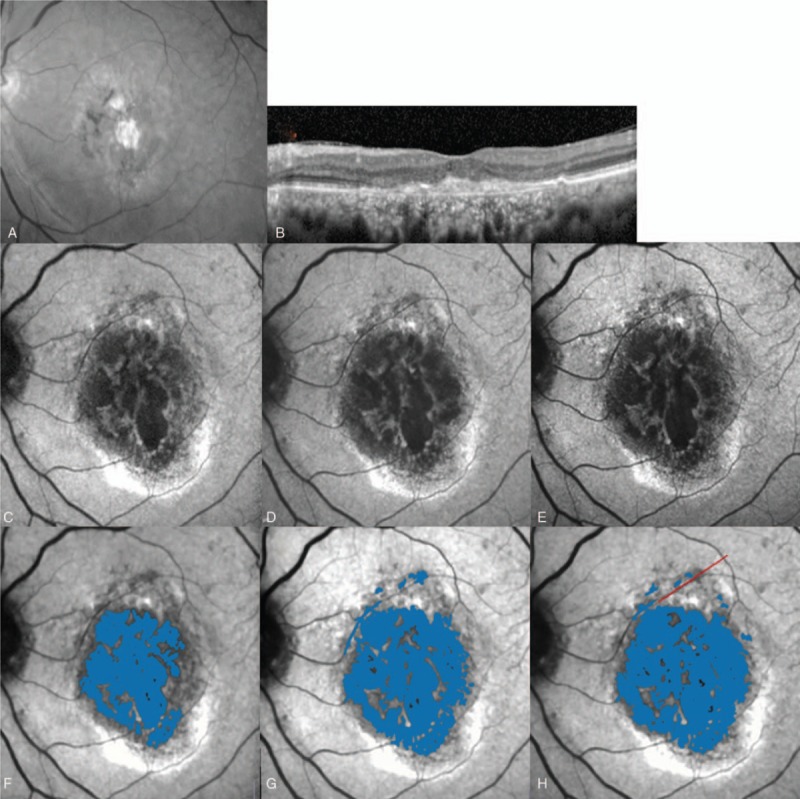
Case 2: A 79-year-old woman with typical age-related macular degeneration who was placed in the placebo group. The patient had received 18 prior intravitreal ranibizumab injections, 1 of which was combined with photodynamic therapy. A gray-scale fundus photograph (A) showing a fibrous scar surrounding retinal pigment epithelial (RPE) atrophy. An optical coherence tomography image (B) shows no exudative lesion. Fundus autofluorescence photographs at baseline (C), 24 weeks (D), and 54 weeks (E) showing the hypofluorescent macular atrophy lesion. The area of macular atrophy (shaded blue) was 6.1 mm^2^ at baseline (F), 7.3 mm^2^ at 24 weeks (G), and 8.4 mm^2^ at 54 weeks (H). The enlargement rate of macular atrophy was 38%.

A 79-year-old woman with typical AMD was placed in the placebo group (Fig. 5). She had 1 prior PDT treatment and 18 prior IVR. At the time of enrollment, the subject had a dry macula, but macular atrophy was apparent on the gray-scale fundus photograph (Fig. 5A), and RPE loss was observed on OCT images (Fig. 5B). To quantify macular atrophy, FAF photographs were taken at baseline (Fig. 5C, F), 24 weeks (Fig. 5D, G), and 54 weeks (Fig. 5E, H). The enlargement rate of macular atrophy was 38% over the 54-week study.

### 3.6 Adverse events

Adverse events were observed in 3 patients during this clinical trial, and all of these patients were removed from the study (Table 4). In the IU group, 1 subject experienced eye irritation and 1 subject died from a myocardial infarction. In the placebo group, 1 subject experienced lower back pain. One subject withdrew from the study for personal reasons, without adverse event. Of the 3 adverse events, only eye irritation was considered possibly related to topical IU use.

**Table 4:**
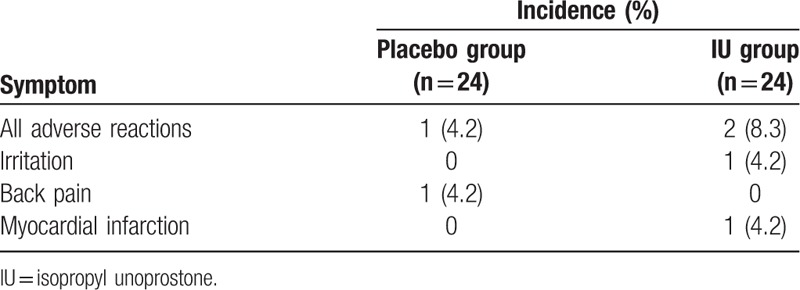
List of adverse drug reactions.

## 4 Discussion

The results of this prospective, placebo-controlled, pilot trial showed that topical IU significantly slowed macular atrophy progression over the 54 weeks that the medication was used. Although, the area of macular atrophy was not significantly different between the IU and placebo groups at any point examined, the average area of macular atrophy in the 2 groups became progressively farther apart over the 54-week study (21 ± 15% in the IU group, and 111 ± 96% in the placebo group). However, the enlargement rate of macular atrophy was significantly different between the IU and placebo groups, suggesting that topical administration of 0.15% IU may slow macular atrophy progression. Moreover, the mean logMAR BCVA improved in the IU group, but it slightly declined in the placebo group at 54 weeks.

A previous study showed that instillation of 3 to 4 drops of 0.12% IU led to a partial mitigation of ET-1-induced vasoconstriction in the human choroid.^[17,32]^ These findings indicate that topical administration of IU may also significantly increase blood flow in the retinal and choroidal circulations in both animals and humans.^[32–34]^ This may be helpful to patients with AMD because prior studies have suggested that inadequate choroidal perfusion and/or choroidal microarchitecture ischemia may lead to hypoxia and ischemia in the overlying RPE.^[7,8]^ Given that maintenance of the RPE and outer retina is so important for good vision,^[35]^ the choroid is also considered to play an important role in visual acuity. The choroidal circulation provides nutrients to and removes metabolic wastes from the RPE and outer retina.^[27–29]^ Thus, an impaired choroidal circulation likely disrupts normal retinal function, leading to visual deterioration. It has been well-documented that choroidal flow and choriocapillaris volume are negatively correlated with aging.^[36]^ Because administration of topical IU has been shown to rescue both RPE and photoreceptor function, it may prevent macular atrophy progression and/or enlargement.^[8,23,24]^

The issue of VEGF-dependent ocular homeostasis has yet to be examined clinically, but preclinical data suggest that VEGF_121_ may be an essential retinal neuroprotectant during ischemic conditions.^[37]^ Moreover, some believe that pan-VEGF blockade, particularly VEGF_121_ blockade, is responsible for macular atrophy enlargement in eyes with AMD and, ultimately, poor visual prognoses.^[38,39]^ According to the results based on 7-year outcomes of eyes treated with ranibizumab for AMD, macular atrophy, with a mean area of 9.4 mm^2^, was detected on FAF images in 98% of eyes.^[39]^ This study also determined that the atrophic area was significantly correlated with a poor visual outcome. The development of macular atrophy leading to RPE atrophy may be related to the frequency with which pan-VEGF-A blockade treatments are administered.^[38,39]^ This could be a serious issue in maintaining good vision in patients with exudative AMD treated with anti-VEGF agents, since follow-up loss was very important in this study, likely due to the duration of the follow-up and comorbidities of patients.

There have been several reports stating that damage to the RPE was observed after PDT in eyes with classic choroidal neovascularization (CNV) in young patients or secondary CNV in pathologic myopia.^[40,41]^ Although a medical history of PDT was not correlated with enlargement of macular atrophy in our study, the relationship between PDT treatment and the development of macular atrophy in AMD is impossible to deny. Furthermore, because there were significant correlations between the subtype of AMD and retinal angiomatous proliferation in the area of macular atrophy, this treatment might be effective in each genotype, particularly, retinal angiomatous proliferation (Table 3). It is possible that treating eyes with macular atrophy with topical 0.15% IU may slow or prevent vision loss in AMD patients.

In summary, our results show that topical 0.15% IU applied 3 times per day may be a useful treatment for progressive macular atrophy secondary to exudative AMD. However, considering the heterogeneity of the patients included in this study, the sample size was too small. Unfortunately, there are no current treatment options for this condition. Therefore, the effect of topical IU on macular atrophy and vision should be further investigated in larger trials with longer follow-up periods.

## References

[1] Klein R Meuer SM Knudtson MD The epidemiology of progression of pure geographic atrophy: the Beaver Dam Eye Study. Am J Ophthalmol 2008;146:692–9. 18672224 10.1016/j.ajo.2008.05.050 PMC2612630

[2] Klein R Chou CF Klein BE Prevalence of age-related macular degeneration in the US population. Arch Ophthalmol 2011;129:75–80. 21220632 10.1001/archophthalmol.2010.318

[3] Sunness JS Gonzalez-Baron J Applegate CA Enlargement of atrophy and visual acuity loss in the geographic atrophy form of age-related macular degeneration. Ophthalmology 1999;106:1768–79. 10485549 10.1016/S0161-6420(99)90340-8

[4] Schatz H McDonald HR Atrophic macular degeneration. Rate of spread of geographic atrophy and visual loss. Ophthalmology 1989;96:1541–51. 2587050 10.1016/s0161-6420(89)32694-7

[5] Smith W Assink J Klein R Risk factors for age-related macular degeneration: pooled findings from three continents. Ophthalmology 2001;108:697–704. 11297486 10.1016/s0161-6420(00)00580-7

[6] Rosenfeld PJ Brown DM Heier JS Ranibizumab for neovascular age-related macular degeneration. N Engl J Med 2006;355:1419–31. 17021318 10.1056/NEJMoa054481

[7] Coleman DJ Silverman RH Rondeau MJ Age-related macular degeneration: choroidal ischaemia? Br J Ophthalmol 2013;97:1020–3. 23740965 10.1136/bjophthalmol-2013-303143 PMC3717761

[8] McLeod DS Grebe R Bhutto I Relationship between RPE and choriocapillaris in age-related macular degeneration. Invest Ophthalmol Vis Sci 2009;50:4982–91. 19357355 10.1167/iovs.09-3639 PMC4829357

[9] Martin DF Maguire MG Fine SL Ranibizumab and bevacizumab for treatment of neovascular age-related macular degeneration: two-year results. Ophthalmology 2012;119:1388–98. 22555112 10.1016/j.ophtha.2012.03.053 PMC3389193

[10] Querques G Massamba N Coscas F Choroidal neovascularisation complicating geographic atrophy in age-related macular degeneration. Br J Ophthalmol 2012;96:1479–83. 23077229 10.1136/bjophthalmol-2012-302191

[11] Schmitz-Valckenberg S Brinkmann CK Alten F Semiautomated image processing method for identification and quantification of geographic atrophy in age-related macular degeneration. Invest Ophthalmol Vis Sci 2011;52:7640–6. 21873669 10.1167/iovs.11-7457

[12] Haria M Spencer CM Unoprostone (isopropyl unoprostone). Drugs Aging 1996;9:213–8. [discussion 219–220]. 8877315 10.2165/00002512-199609030-00007

[13] Yamamoto T Kitazawa Y Azuma I Clinical evaluation of UF-021 (Rescula; isopropyl unoprostone). Surv Ophthalmol 1997;41 suppl 2:S99–103. 9154284 10.1016/s0039-6257(97)80015-x

[14] Gunawardena KA Crame N Mertz B Safety of unoprostone isopropyl 0.15% ophthalmic solution in patients with mild to moderate asthma. Ophthalmologica 2003;217:129–36. 12592052 10.1159/000068558

[15] Mizuno K Koide T Shimada S Route of penetration of topically instilled nipradilol into the ipsilateral posterior retina. Invest Ophthalmol Vis Sci 2009;50:2839–47. 19218605 10.1167/iovs.08-2922

[16] Kimura I Shinoda K Tanino T Effect of topical unoprostone isopropyl on optic nerve head circulation in controls and in normal-tension glaucoma patients. Jpn J Ophthalmol 2005;49:287–93. 16075327 10.1007/s10384-004-0208-2

[17] Polska E Doelemeyer A Luksch A Partial antagonism of endothelin 1-induced vasoconstriction in the human choroid by topical unoprostone isopropyl. Arch Ophthalmol 2002;120:348–52. 11879139 10.1001/archopht.120.3.348

[18] Cuppoletti J Malinowska DH Tewari KP Cellular and molecular effects of unoprostone as a BK channel activator. Biochim Biophys Acta 2007;1768:1083–92. 17307133 10.1016/j.bbamem.2006.12.015

[19] Melamed S Neuroprotective properties of a synthetic docosanoid, unoprostone isopropyl: clinical benefits in the treatment of glaucoma. Drugs Exp Clin Res 2002;28:63–73. 12224379

[20] Munemasa Y Kitaoka Y Hayashi Y Effects of unoprostone on phosphorylated extracellular signal-regulated kinase expression in endothelin-1-induced retinal and optic nerve damage. Vis Neurosci 2008;25:197–208. 18442442 10.1017/S095252380808053X

[21] Mukuno H Nakamura M Kanamori A Unoprostone isopropyl rescues retinal progenitor cells from apoptosis in vitro. Curr Eye Res 2004;29:457–64. 15764090 10.1080/02713680490889465

[22] Tsuruma K Tanaka Y Shimazawa M Unoprostone reduces oxidative stress- and light-induced retinal cell death, and phagocytotic dysfunction, by activating BK channels. Mol Vis 2011;17:3556–65. 22219651 PMC3250378

[23] Tawada A Sugawara T Ogata K Improvement of central retinal sensitivity six months after topical isopropyl unoprostone in patients with retinitis pigmentosa. Ind J Ophthalmol 2013;61:95–9. 10.4103/0301-4738.109377 PMC3665054 23514642

[24] Yamamoto S Sugawara T Murakami A Topical isopropyl unoprostone for retinitis pigmentosa: microperimetric results of the phase 2 clinical study. Ophthalmol Ther 2012;1:5. 25135585 10.1007/s40123-012-0005-9 PMC4108136

[25] Falsini B Anselmi GM Marangoni D Subfoveal choroidal blood flow and central retinal function in retinitis pigmentosa. Invest Ophthalmol Vis Sci 2011;52:1064–9. 20861481 10.1167/iovs.10-5964

[26] Vingolo EM Lupo S Grenga PL Endothelin-1 plasma concentrations in patients wth retinitis pigmentosa. Regulat Pept 2010;160:64–7. 10.1016/j.regpep.2009.12.006 20005906

[27] Zarbin MA Current concepts in the pathogenesis of age-related macular degeneration. Arch Ophthalmol 2004;122:598–614. 15078679 10.1001/archopht.122.4.598

[28] Friedman E Oak SM Choroidal microcirculation in vivo. Bibl Anat 1965;7:129–32. 5860720

[29] Grunwald JE Siu KK Jacob SS Effect of sildenafil citrate (Viagra) on the ocular circulation. Am J Ophthalmol 2001;131:751–5. 11384572 10.1016/s0002-9394(00)00944-2

[30] Sunness JS Margalit E Srikumaran D The long-term natural history of geographic atrophy from age-related macular degeneration: enlargement of atrophy and implications for interventional clinical trials. Ophthalmology 2007;114:271–7. 17270676 10.1016/j.ophtha.2006.09.016 PMC2562326

[31] Grunwald JE Pistilli M Ying GS Growth of geographic atrophy in the comparison of age-related macular degeneration treatments trials. Ophthalmology 2015;122:809–16. 25542520 10.1016/j.ophtha.2014.11.007 PMC4372487

[32] Yu DY Su EN Cringle SJ Comparison of the vasoactive effects of the docosanoid unoprostone and selected prostanoids on isolated perfused retinal arterioles. Invest Ophthalmol Vis Sci 2001;42:1499–504. 11381053

[33] Grunwald JE Metelitsina TI Dupont JC Reduced foveolar choroidal blood flow in eyes with increasing AMD severity. Invest Ophthalmol Vis Sci 2005;46:1033–8. 15728562 10.1167/iovs.04-1050

[34] Kojima S Sugiyama T Azuma I [Effect of topically applied isopropyl unoprostone on microcirculation in the human ocular fundus evaluated with a laser speckle microcirculation analyser]. Nippon Ganka Gakkai zasshi 1997;101:605–10. 9256623

[35] Linsenmeier RA Padnick-Silver L Metabolic dependence of photoreceptors on the choroid in the normal and detached retina. Invest Ophthalmol Vis Sci 2000;41:3117–23. 10967072

[36] Grunwald JE Piltz J Patel N Effect of aging on retinal macular microcirculation: a blue field simulation study. Invest Ophthalmol Vis Sci 1993;34:3609–13. 8258519

[37] Nishijima K Ng YS Zhong L Vascular endothelial growth factor-A is a survival factor for retinal neurons and a critical neuroprotectant during the adaptive response to ischemic injury. Am J Pathol 2007;171:53–67. 17591953 10.2353/ajpath.2007.061237 PMC1941589

[38] Saint-Geniez M Kurihara T Sekiyama E An essential role for RPE-derived soluble VEGF in the maintenance of the choriocapillaris. Proc Natl Acad Sci U S A 2009;106:18751–6. 19841260 10.1073/pnas.0905010106 PMC2774033

[39] Rofagha S Bhisitkul RB Boyer DS Seven-year outcomes in ranibizumab-treated patients in ANCHOR, MARINA, and HORIZON: a multicenter cohort study (SEVEN-UP). Ophthalmology 2013;120:2292–9. 23642856 10.1016/j.ophtha.2013.03.046

[40] Postelmans L Pasteels B Coquelet P Severe pigment epithelial alterations in the treatment area following photodynamic therapy for classic choroidal neovascularization in young females. Am J Ophthalmol 2004;138:803–8. 15531316 10.1016/j.ajo.2004.06.033

[41] Krebs I Binder S Stolba U Choroidal neovascularization in pathologic myopia: three-year results after photodynamic therapy. Am J Ophthalmol 2005;140:416–25. 16139000 10.1016/j.ajo.2005.03.050

